# Effects of postural threat on the scaling of anticipatory postural adjustments in young and older adults

**DOI:** 10.3389/fnhum.2023.1267093

**Published:** 2023-09-28

**Authors:** Angel L. Phanthanourak, Allan L. Adkin, Mark G. Carpenter, Craig D. Tokuno

**Affiliations:** ^1^Department of Kinesiology, Brock University, St. Catharines, ON, Canada; ^2^School of Kinesiology, University of British Columbia, Vancouver, BC, Canada

**Keywords:** anticipatory postural adjustment, postural threat, aging, balance control, electromyography, center of pressure

## Abstract

**Introduction:**

The ability to scale anticipatory postural adjustments (APAs) according to the predicted size of the upcoming movement is reduced with aging. While age-related changes in central set may be one reason for this effect, an individual’s emotional state might also contribute to changes in anticipatory postural control. Therefore, the purpose of this study was to determine whether an altered emotional state, as elicited through postural threat, alters the scaling of APAs during a handle pull movement in young and older adults. It was hypothesized that the presence of postural threat would lead to more homogenous APAs (i.e., less scaling of APAs) across a range of pulling forces.

**Methods:**

Young (*n* = 23) and older adults (*n* = 16) stood on top of a force plate that was mounted to a motorized platform. From this position, participants performed a series of handle pull trials without (no threat) or with (threat) the possibility of receiving a postural perturbation in the form of an unpredictable surface translation. Handle pulls were performed at force levels between 50 and 90% of maximum force. For each trial, the magnitude and timing of the APA were quantified from center of pressure (COP) recordings as well as electromyographic (EMG) activity of the soleus and medial gastrocnemius. The scaling of APAs with respect to force exertion was then determined through regression analyses and by comparing APAs during pulls of lower versus higher force.

**Results and discussion:**

As evidenced by their smaller slope of the regression line between various dependent measures (i.e., COP velocity, soleus EMG onset latency, and soleus EMG amplitude) and the pulled forces, older adults demonstrated less scaling of APAs than the young. However, increases in arousal, anxiety and fear of falling due to postural threat, only minimally altered the scaling of APAs. Regardless of age, the slope of the regressions for none of the measures were affected by threat while only the soleus and medial gastrocnemius EMG onsets demonstrated significant force × threat interaction effects. These results suggest that the decreased ability to scale APAs with aging is unlikely to be due to changes in emotional state.

## 1. Introduction

Anticipatory postural adjustments (APAs) involve patterns of postural muscle activity generated by the central nervous system (CNS) in preparation of an upcoming voluntary movement. The resulting deviations to the center of pressure (COP) and center of mass (COM) serve two purposes. First, APAs assist with the initiation of an impending movement by destabilizing the COM and propelling the body into motion. This is commonly observed during the rise-to-toes movement, where the initial activation of the tibialis anterior (TA) and the backward COP displacement act to push the COM forward and towards the toes (Adkin et al., 2002; Phanthanourak et al., 2016). Second, and of relevance to this study, APAs serve to proactively counteract the forces that are expected from an upcoming movement. For example, prior to pulling on a handle towards the body, individuals first activate the triceps surae to displace the COP forwards, which causes the COM to shift backwards prior to the start of the handle pull (Cordo and Nashner, 1982; Lee et al., 1990; Weeks, 1994). Once the APA has been initiated, the prime movers (i.e., biceps brachii and posterior deltoids) are then activated to exert the appropriate amount of force onto the handle (Cordo and Nashner, 1982; Lee et al., 1990).

Regardless of its purpose, APAs are often scaled in magnitude and timing according to the predicted size of the upcoming movement (Bouisset and Do, 2008). For example, larger and earlier bursts of preparatory muscle activity facilitate a greater forward COP displacement in advance of a stronger handle pull (Lee et al., 1990; Weeks, 1994). This scaling of the APA is important because it enables the COM to start farther away from the boundary of the base of support and ensures a greater margin of stability during more forceful and de-stabilizing movements (Massion, 1992; Weeks, 1994). Interestingly, older adults do not scale their APAs to the same extent as young adults and consequently, this may be a potential contributor to their increased risk of falls (Weeks, 1994; Kubicki et al., 2012).

The reduced scaling of APAs associated with aging may be related to an impaired ability to adequately predict the upcoming postural instability (i.e., central set). This is because the size and timing of APAs are programmed according to the expected rather than the actual characteristics of the upcoming perturbation (Toussaint et al., 1998). Although central set may be reduced with aging (Horak et al., 1989), an individual’s emotional state, independent of aging, can also affect central set. For example, when individuals experience an increase in fear of falling and anxiety, this affects their ability to prepare for an upcoming motor response and consequently, balance control (Brown et al., 2002). Therefore, it is not clear whether the altered scaling of APAs associated with older adults is directly related to aging or is influenced by changes in emotional state.

To dissociate the contributions of aging and emotion on anticipatory balance control, previous studies have incorporated a postural threat paradigm. When young adults perform a movement while standing at the edge of an elevated height or while anticipating a potential surface translation, these postural threat conditions cause individuals to experience elevated levels of physiological arousal, anxiety and fear of falling (Adkin et al., 2002; Phanthanourak et al., 2016; Bax et al., 2020). This leads to alterations to the size and timing of APAs, though the specific changes vary depending on the form of postural threat and the movement being performed. For example, when individuals perform a heel raise as fast as possible under of the threat of a postural perturbation, larger and faster APAs are observed (Phanthanourak et al., 2016; Bax et al., 2020). In contrast, when the same movement is performed at the edge of a raised platform to induce postural threat, the APAs become smaller than when the movement is performed at a lower height (Adkin et al., 2002). Lastly, APAs associated with gait initiation are disproportionately larger when the movement is performed on an elevated compared to ground height (Ellmers et al., 2020).

It is evident from these studies that an altered emotional state elicited through the presence of postural threat affects APAs. However, these studies have only assessed APAs during movements performed at a single (i.e., maximal) speed and by young adults. It is not known if postural threat also influences the scaling of APAs, whereby larger movements requiring larger APAs might be differentially affected by the threat. Further, it is possible that the effects of threat on APAs might be differentially affected by age. Elevated levels of anxiety and fear of falling due to postural threat increase the attentional demands associated with gait (Gage et al., 2003). But since older adults have a more limited attentional capacity (Huxhold et al., 2006), these increased attentional demands may result in greater impairments in the ability to scale their APA. This could explain why fearful older adults require more time to generate APAs when dual-tasking compared to their non-fearful, age-matched counterparts (Uemura et al., 2012).

Therefore, the aim of this study was to determine whether postural threat alters the scaling of APAs during a handle pull movement in young and older adults. This study relied on a handle pull movement, instead of the heel raise, leg raise, or gait initiation tasks used in previous threat studies, to allow participants to perform the movement at different force levels and consequently, with different APA requirements. Both young and older adults were examined to corroborate previous age-related differences in anticipatory postural control and to determine whether a heightened emotional state attenuates or magnifies any age-related differences in APA scaling. If a heighted state of emotion is responsible for the ability to plan for upcoming movements, it was hypothesized that the presence of postural threat would lead to more homogenous APAs (i.e., less scaling of APAs) across a range of forces in both young and older adults.

## 2. Methods

### 2.1. Participants

Twenty-three young adults and 16 community-dwelling older adults with no known neuromuscular or orthopedic disorders or injuries that could affect their balance participated in this study. Participant characteristics for each group are presented in Table 1. All participants provided written informed consent prior to their involvement in the study. All experimental procedure were conducted in accordance with the university research ethics board and the Declaration of Helsinki.

**Table 1:** Participant demographics for the young and older adult groups.

	Young adults	Older adults
Age (y)	23.6 ± 2.4	69.8 ± 6.0
Sex	9 F, 14 M	7 F, 9 M
Height (cm)	170.6 ± 10.3	172.0 ± 8.5
Mass (kg)	69.2 ± 10.2	78.6 ± 14.2
ABC (/100)	95.1 ± 4.8	94.2 ± 4.7

Data are presented as mean ± 1 SD. ABC refers to the activities-specific balance confidence questionnaire, which has a maximum score of 100.

### 2.2. Experimental setup

Pairs of surface electrodes (32 mm diameter, 5 mm interelectrode distance, Kendall Meditrace 200, Mansfield, MA, United States) were placed on the skin over the right biceps brachii, soleus (SOL) and medial gastrocnemius (MG). Surface electromyographic (EMG) recordings from the biceps brachii were used to monitor the handle pull, while EMG activity from the SOL and MG were used to quantify the APA (Cordo and Nashner, 1982; Inglin and Woollacott, 1988). Although Lee et al. (1990) also observed EMG activity from the tibialis anterior in advance of a handle pull, this muscle was found to be recruited in a smaller proportion of trials, particularly at lower pulling forces, and with a larger between-subject variability in EMG onset latencies than the gastrocnemius. Therefore, this study focused solely on the activation of the calf muscles when quantifying the APA. A single reference electrode (same material as above) was placed on the lateral aspect of the right knee. These skin sites were shaved, cleansed with alcohol, and lightly abraded with a conductive gel (NuPrep, Weaver and Company, Aurora, CO, United States) prior to electrode placement. An additional two electrodes (EL-507, BIOPAC Systems Inc., United States) were placed on the participant’s non-dominant hand and connected to an electrodermal activity unit (EDA100C, BIOPAC Systems Inc., Goleta, CA, United States).

### 2.3. Experimental protocol

**Figure 1:**
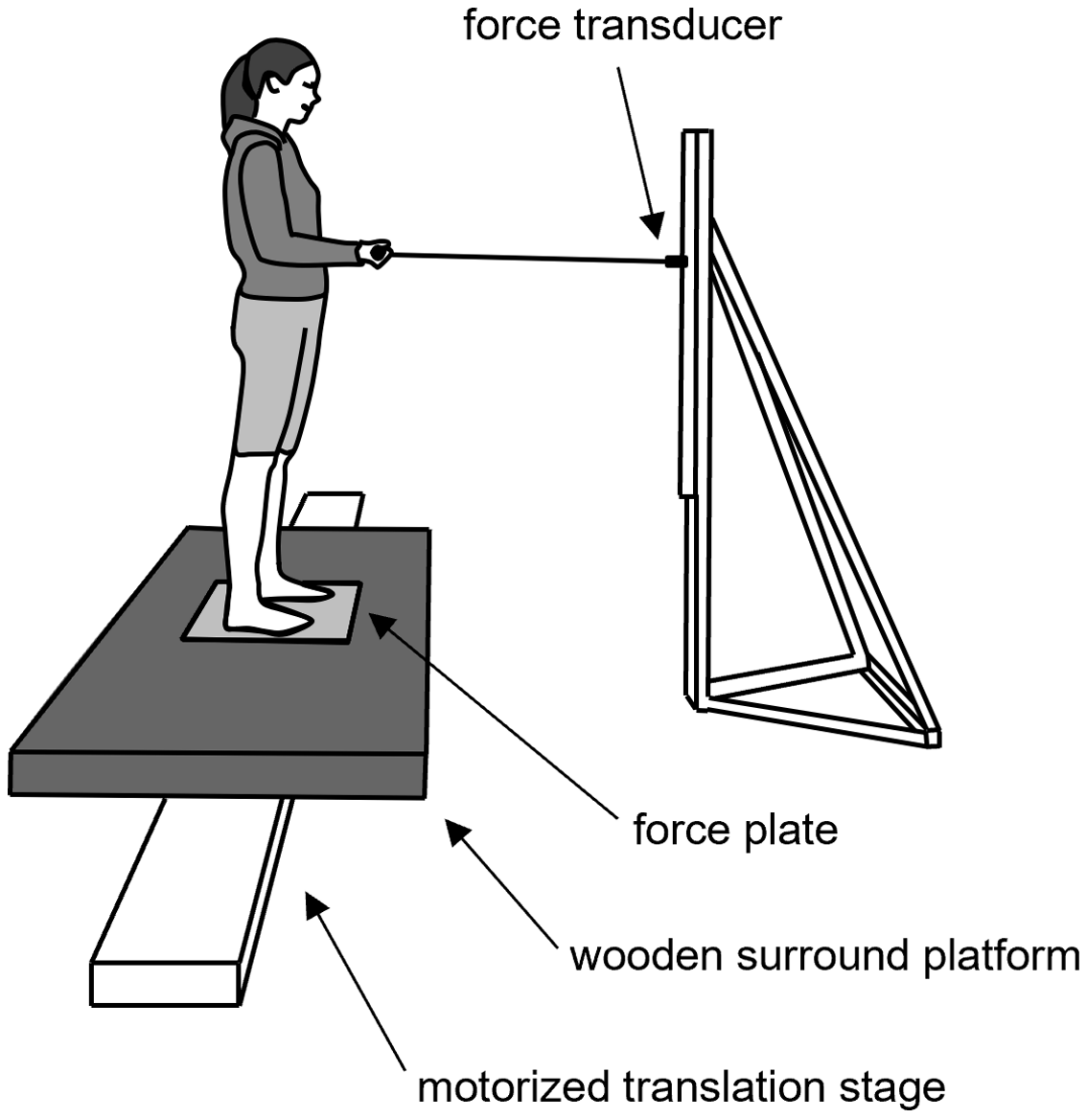
Illustration of the experimental setup. Participants stood with their feet positioned no wider than their foot length apart on a force platform that was flush with a 0.9 m × 1.6 m wooden surround platform and attached to a 4.3 m motorized translation stage. When instructed, participants pulled on the handle bar that was attached to strain gauge transducer via a steel wire. Participants held the handle bar with their forearms parallel to the ground, their elbows flexed at ~90 degrees and with their hands placed shoulder width apart.

Participants stood barefoot and with their feet positioned no wider than their foot length apart on a force plate (0.46 m × 0.51 m, AMTI, OR6-7-2000, Watertown, MA, United States) that was flush with a wooden platform fixed to a motorized translation stage (H2W Technologies Inc., Valencia, CA, United States) (Figure 1). Participants wore a body harness that was attached to an overhead track to prevent the participant from experiencing a fall. From this standing position, participants held a steel handle bar that was attached to an anchored strain gauge transducer via a steel wire (Figure 1). The bar and strain gauge transducer were adjusted in height so that the connecting wire, as well as the participant’s forearms were parallel to the ground when the participant’s elbows were flexed at ~90 degrees. Participants gripped the bar with their hands placed shoulder width apart.

The participant’s maximum pulling force (F_max_) was determined by having them pull as hard as possible on the handle bar using their biceps brachii, and without stepping or losing their balance. The largest peak force from three trials was deemed F_max_. Participants then completed a practice condition consisting of 12 handle pull trials. Each handle pull trial commenced with the researcher verbally indicating the target force, as a percentage of the participant’s F_max_, for the upcoming pull. The target force was between 50 to 100% of their F_max_, in 10% increments, and was presented in a pseudo-random order. Participants were then presented with an auditory “warning” tone followed 1–4 s later by a higher pitched “go” tone. Upon hearing the “go” tone, participants pulled onto the handle with the instructed amount of force. The force was maintained for ~1 s before participants relaxed and released the pull. Visual feedback of the participant’s force was provided online throughout the pull.

Participants then completed the No Threat condition, which consisted of 36 handle pull trials. Participants pulled at a pre-determined force level that was between 50 and 90% of F_max_ following the same auditory tones as the practice conditions. During the pull, participants were not provided any visual feedback of their force to avoid any over-correcting that may occur to achieve the stated goal force. Nonetheless, participants were instructed that the goal force was simply a guide and that inaccurate handle pulls would not be discarded. Lastly, participants were informed that the platform that they were standing on would remain stationary and locked in place for the entirety of the condition. Rest periods were provided to minimize fatigue.

Following the No Threat condition, participants completed the Threat condition, which consisted of 45 trials. Like the No Threat condition, participants were instructed to pull at a pre-determined force level without any feedback of their force. However, participants were also informed that the platform that they were standing on may or may not move at any time following the “warning” tone. The potential medio-lateral surface translation had a displacement of 0.25 m in the leftward or rightward direction, a peak velocity of 0.7 m/s, and a peak acceleration of 1.6 m/s^2^. Previous studies have found that these platform parameters elicited a threat response that was large enough to induce changes to both static and anticipatory postural control (Phanthanourak et al., 2016; Johnson et al., 2019; Bax et al., 2020). For 15 of the 45 trials, the platform remained stationary and the “warning” tone was followed by the “go” tone, cuing participants to complete the handle pull at the instructed force. For the remaining 30 trials, the platform translation occurred at any time before the “go” tone until 3 s after the “go” tone. In response to the platform translation, participants were instructed to recover their balance however they deemed necessary. Participants were unaware of the timing of the surface translations. Only trials where the pulling action was not influenced by the surface translation (i.e., trials without a perturbation or those that occurred sufficiently after the pull was completed) were included for analyses.

### 2.4. Data collection and analyses

#### 2.4.1. Physiological arousal and psycho-social measures

Electrodermal activity (EDA) was collected from the participants’ non-dominant hand. The EDA signal was sampled at 1,000 Hz (micro1401, Cambridge Electronics Design, Cambridge, United Kingdom) and filtered offline using a second-order Butterworth low-pass (10 Hz) filter (Adkin et al., 2002). For each handle pull trial, the average EDA during the 2 s immediately prior to the “go” tone was calculated (Phanthanourak et al., 2016). Ensemble averages were calculated for each condition.

Prior to each experimental condition, participants rated, on a scale from 0% (not at all confident) to 100% (completely confident), their confidence in maintaining their balance and avoiding a fall for the upcoming condition (Adkin et al., 2002; Davis et al., 2009; Phanthanourak et al., 2016). At the end of each experimental condition, participants rated, on a scale from 0% (no fear) to 100% (extremely fearful), how fearful of falling they felt when completing the handle pulls (Adkin et al., 2002; Davis et al., 2009; Phanthanourak et al., 2016). Participants also completed a 16-item state anxiety survey modified from Smith et al. (1990). Each item was scored on a scale of 1–9 (1 = “I did not feel this at all,” 9 = “I felt this extremely”), and the 16 scores were summed (Carpenter et al., 2006; Davis et al., 2009).

#### 2.4.2. Pulling force

A strain gauge transducer recorded the pulling force exerted onto the handle at a sampling rate of 1,000 Hz (micro1401, Cambridge Electronics Design, Cambridge, United Kingdom). For each handle pull trial, baseline force was defined as the average force activity that occurred during the 200 ms interval following the “warning” tone. Next, pulling force onset was defined to occur when the force signal was 2 standard deviations (SDs) greater the baseline force. Following force onset, the peak force was determined and normalized to the participant’s F_max_.

#### 2.4.3. Surface electromyography

For each handle pull trial, SOL and MG EMG onset latencies were visually determined by the researcher. The onset latency was determined as the point when the EMG activity was first larger than baseline EMG activity for at least 50 ms, where baseline EMG activity was considered to occur during the 250 ms interval following the “warning” tone (Adkin et al., 2002; Phanthanourak et al., 2016). The EMG amplitude of each muscle was then quantified as the root mean square (RMS) over the 250 ms interval following EMG onset of the respective trial (Adkin et al., 2002). EMG data were rectified and filtered using a fourth order, 50 Hz low-pass Butterworth filter prior to calculating the EMG amplitudes.

#### 2.4.4. Center of pressure

COP was calculated from the force and moment signals obtained from the force plate (AMTI, OR6-7-2000, Watertown, MA, United States) on which the participants stood. The force and moment signals were sampled at a rate of 1,000 Hz (micro1401, Cambridge Electronics Design, Cambridge, United Kingdom). COP analyses were limited to the A-P direction because the pulling movement and the associated APA only occur in this direction.

For each handle pull trial, baseline COP was determined as the mean COP signal during the 200 ms interval following the “warning” tone. COP onset was then determined as the point when the COP signal exceeded 2 SDs above baseline COP. COP displacement at pulling onset was calculated as the change in COP position from COP onset to pulling force onset. Lastly, peak COP velocity for each handle pull trial was determined by differentiating the COP signal and finding the maximum positive value.

### 2.5. Statistical analyses

The effect of postural threat on physiological arousal and psycho-social measures were determined by performing 2 (age) × 2 (threat) mixed model ANOVAs on the average EDA activity as well as the reported levels of balance confidence, fear of falling and anxiety.

To assess the effect of postural threat on the scaling of APAs, several measures relating to the APA were considered. APA magnitude was quantified by the COP displacement at pulling force onset, the peak COP velocity as well as the SOL and MG EMG amplitudes. APA timing was represented by the COP onset latency as well as the SOL and MG EMG onset latencies, all of which were reported relative to the pulling force onset. Although the distribution of target forces was the same for all participants and between the No Threat and Threat conditions, differences in the actual pulling forces were observed (see Results). Therefore, each subject’s COP, EMG and pulling force data were linearly transformed to a standardized *z*-score using the equation (trial data – mean value for a given participant and threat condition) divided by the standard deviation for a given participant and threat condition. The transformation was applied to the No Threat and Threat conditions separately. A linear regression line was then applied to the transformed data for each subject and threat condition, with pulling force as the independent variable and each of the COP and EMG measures as the dependent measure. Lastly, the slope of each regression line was determined to quantify the degree of APA scaling as a function of pulling force. Separate 2 (group: young vs. older adults) × 2 (threat: No Threat vs. Threat) mixed model ANOVAs were conducted on the obtained slope values for each APA measure using commercially available software (IBM SPSS Statistics 22, Chicago, IL, United States). Post-hoc Bonferroni-corrected paired *t*-tests were conducted to follow up on any significant interaction effects. Significance for all tests was set to *p* < 0.05.

Transforming the COP and EMG data can account for some, but not all, of the differences in the observed range of pulling forces between individuals, age groups and threat conditions. Therefore, secondary analyses were performed to compare APAs between the No Threat and Threat conditions and between the young and older adults over the same range of pulling forces. This was achieved by creating a low (50–70 %MVC) and high (80–100 %MVC) force condition. Trial data from each participant were only included for analysis if the actual pulling force for a given trial occurred within one of these two force conditions and a participant’s average was only considered for statistical analysis if there were a minimum of three trials for each of the No Threat and Threat conditions at both the low and high force bins. These specific force bins (i.e., 50–70 %MVC and 80–100 %MVC) and minimum trial requirements were chosen based on the distribution of pulled forces, where it optimized the number of participants and trial data that could be analyzed (e.g., few participants pulled at forces less than 50 %MVC for both threat conditions) and ensured some separation in pulled forces between the low and high force conditions. This method resulted in 12.2 ± 6.7 trials being included for each threat-force condition per participant and data from 17 young adults and 10 older adults being included for statistical analyses.

The same APA magnitude and timing measures were examined for this secondary analysis as were done for the regression analysis. However, this data was now analyzed using three-way mixed model ANOVAs, with age (young vs. older adults) as the randomized group factor, and threat (No Threat vs. Threat) and pulling force (low vs. high force) as the repeated factors (IBM SPSS Statistics 22, Chicago, IL, United States). Post-hoc Bonferroni-corrected independent or paired *t*-tests were conducted where appropriate. Significance for all tests was set to *p* < 0.05. Data are presented as the mean ± one SD.

## 3. Results

### 3.1. Pulling force

Although the distribution of target forces was the same for all participants and between the No Threat and Threat conditions, differences in the actual pulling forces were observed. Both the mean (F_1,37_ = 19.10; *p* < 0.001) and standard deviation (F_1,37_ = 7.26; *p* = 0.011) of forces were influenced by an age × threat interaction effect. Post-hoc *t*-tests indicated that for the young adults, there was a trend for harder pulls during the Threat (64.3 ± 5.9 %MVC) compared to the No Threat (62.5 ± 5.4 %MVC) condition (t_22_ = 1.92; *p* = 0.067). However, a larger difference in mean force was observed in the older adults, where they pulled harder during the Threat (71.2 ± 8.6 %MVC) compared to the No Threat (61.4 ± 10.2 %MVC) condition (t_15_ = 5.58; *p* < 0.001). When the between-trial variability in pulling force within each condition was considered, no differences in the dispersion of forces (i.e., SD) were observed in the young adults (No Threat: 14.3 ± 2.2 %MVC; Threat: 14.3 ± 2.3 %MVC; *p* = 0.991). In contrast, older adults pulled with a smaller dispersion of forces during the Threat (10.8 ± 2.4 %MVC) compared to the No Threat (13.1 ± 2.3 %MVC) conditions (t_15_ = 3.82; *p* = 0.002).

### 3.2. Physiological arousal and psychosocial measures

The effectiveness of the presented postural threat was confirmed through the measurement of physiological arousal (i.e., EDA) and psychosocial responses (Table 2). The presence of postural threat resulted an increased EDA (threat main effect; F_1,37_ = 8.88; *p* = 0.005), an increased fear of falling (threat main effect; F_1,37_ = 38.33; *p* < 0.001) and a decreased balance confidence (threat main effect; F_1,37_ = 42.73; *p* < 0.001). Perceived anxiety was influenced by an age × threat interaction effect (F_1,37_ = 9.98; *p* = 0.012), where young adults reported more anxiety in the Threat compared to No Threat condition (*p* < 0.001), but older adults’ perceived anxiety did not change with threat (*p* = 0.105). Lastly, a main effect of age was observed for EDA, where across both threat conditions, EDA was greater in young (5.02 ± 1.90 μS) compared to older adults (3.39 ± 2.02 μS) (F_1,37_ = 6.61; *p* = 0.014).

**Table 2:** Mean ± 1 SD electrodermal activity, balance confidence, fear of falling, and perceived anxiety for the No Threat and Threat experimental conditions.

	Young adults		Older adults	
	No Threat	Threat	No Threat	Threat
EDA (μS)	4.70 ± 1.92	5.34 ± 2.01	3.21 ± 1.83	3.58 ± 2.33
Balance confidence (/100)	98.9 ± 3.0	65.0 ± 26.5	99.7 ± 1.3	72.8 ± 31.1
Fear of falling (/100)	0.7 ± 2.3	43.3 ± 32.6	3.4 ± 12.5	28.0 ± 34.1
Perceived anxiety (/154)	27.6 ± 12.1	53.3 ± 27.0	18.9 ± 5.7	27.3 ± 16.7

EDA, electrodermal activity.

### 3.3. Regression analyses

When the slope of the regression lines between the various dependent measures and the pulled force were analyzed, a main effect of age was observed for COP velocity (F_1,37_ = 7.14, *p* = 0.011), SOL EMG onset (F_1,37_ = 5.87, *p* = 0.020) and SOL EMG amplitude (F_1,37_ = 9.61, *p* = 0.004) (Table 3). For all three measures, the scaling (slope) of APAs was less, by 37.2–47.2%, in older compared to young adults. A trend towards a difference in the scaling of the MG EMG onset latency between age groups was also noted (F_1,37_ = 3.84, *p* = 0.055), with 36.6% less scaling in the older compared to young adults.

**Table 3:** Mean (1 SD) slope of the regression line between each dependent measure and the pulled force for the No Threat and Threat experimental conditions.

	Young adults		Older adults	
	No Threat	Threat	No Threat	Threat
COP displacement^b^	0.57 (0.22)	0.63 (0.19)	0.61 (0.27)	0.56 (0.21)
COP onset	−0.36 (0.23)	−0.38 (0.22)	−0.33 (0.31)	−0.35 (0.18)
COP velocity^a^	0.43 (0.25)	0.42 (0.21)	0.29 (0.31)	0.25 (0.19)
SOL EMG onset^a^	−0.33 (0.17)	−0.27 (0.22)	−0.16 (0.25)	−0.16 (0.18)
MG EMG onset	−0.36 (0.18)	−0.32 (0.25)	−0.21 (0.26)	−0.22 (0.24)
SOL EMG amplitude^a^	0.33 (0.19)	0.36 (0.19)	0.20 (0.22)	0.20 (0.16)
MG EMG amplitude	0.21 (0.24)	0.16 (0.23)	0.14 (0.25)	0.18 (0.20)

Data were linear transformed using standardized *z*-scores and therefore, all slopes are unitless. Negative slope values indicate earlier onsets with increasing force.

a Indicates a significant main effect of age.

b Indicates a significant age × threat interaction effect.

COP, center of pressure; EMG, electromyography; SOL, soleus; MG, medial gastrocnemius.

**Figure 2:**
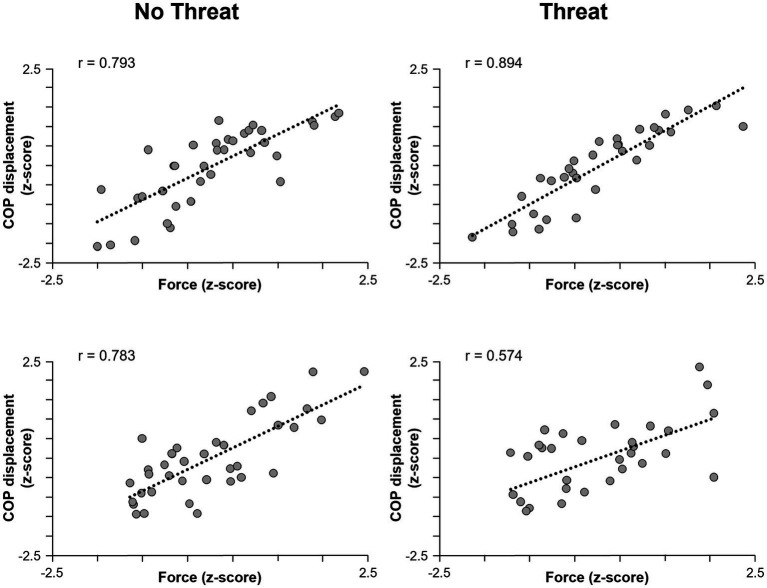
Scatterplots of pulling force (horizontal axis) and COP displacement (vertical axis) for one representative young adult (top row) and one older adult (bottom row) participant. Data for the No Threat and Threat conditions are plotted in the left and right columns, respectively. The regression lines indicate the slope of the relationship. COP, center of pressure.

The only dependent measure that was influenced by the presence of postural threat was COP displacement, where an age × threat interaction effect was observed (F_1,37_ = 4.25, *p* = 0.046) (Figure 2). Although the presence of postural threat appeared to increase the scaling of COP displacement in young adults but decrease the scaling in young adults, post-hoc analyses revealed no significant differences between groups or threat conditions.

Exploratory analyses were also performed to investigate whether these findings could be attributed to participants habituating to the platform perturbation and being less threatened over time. Data from the Threat condition was divided into an early (1st quartile of trials) and late (4th quartile of trials) and separate regression analyses were computed on the early and late trials. The computed slope values were then inputted into separate 2 (age) × 2 (time: early vs. late) ANOVAs for each measure. These analyses revealed that only COP velocity was affected an age × time interaction effect (F_1,37_ = 4.68; *p* = 0.037). However, when the data were separated by age, post-hoc tests did not reveal any significant differences in the scaling of the COP velocity between the early compared to the late trials for both the young (*p* = 0.260) and older adults (*p* = 0.070). All other main and interaction effects relating to time were not significant.

### 3.4. Secondary analyses

Since the data were limited to trials with a pulling force between 50 and 70%, and 80 and 100%MVC, it is not surprising that there were few differences in pulling force between age groups and threat conditions. The three-way ANOVA revealed an age × force × threat interaction effect (F_1,25_ = 4.63; *p* = 0.041). Post-hoc analyses indicated that pulling force was only different between the No Threat and Threat conditions of the low force condition in older adults. However, since the actual magnitude of difference between conditions was only 2.81 %MVC, from 60.1 %MVC to 62.9 %MVC during the No Threat and Threat conditions, respectively, it is unlikely that this would have significantly influenced the APA data.

#### 3.4.1. The effect of pulling force and age on APAs

**Figure 3:**
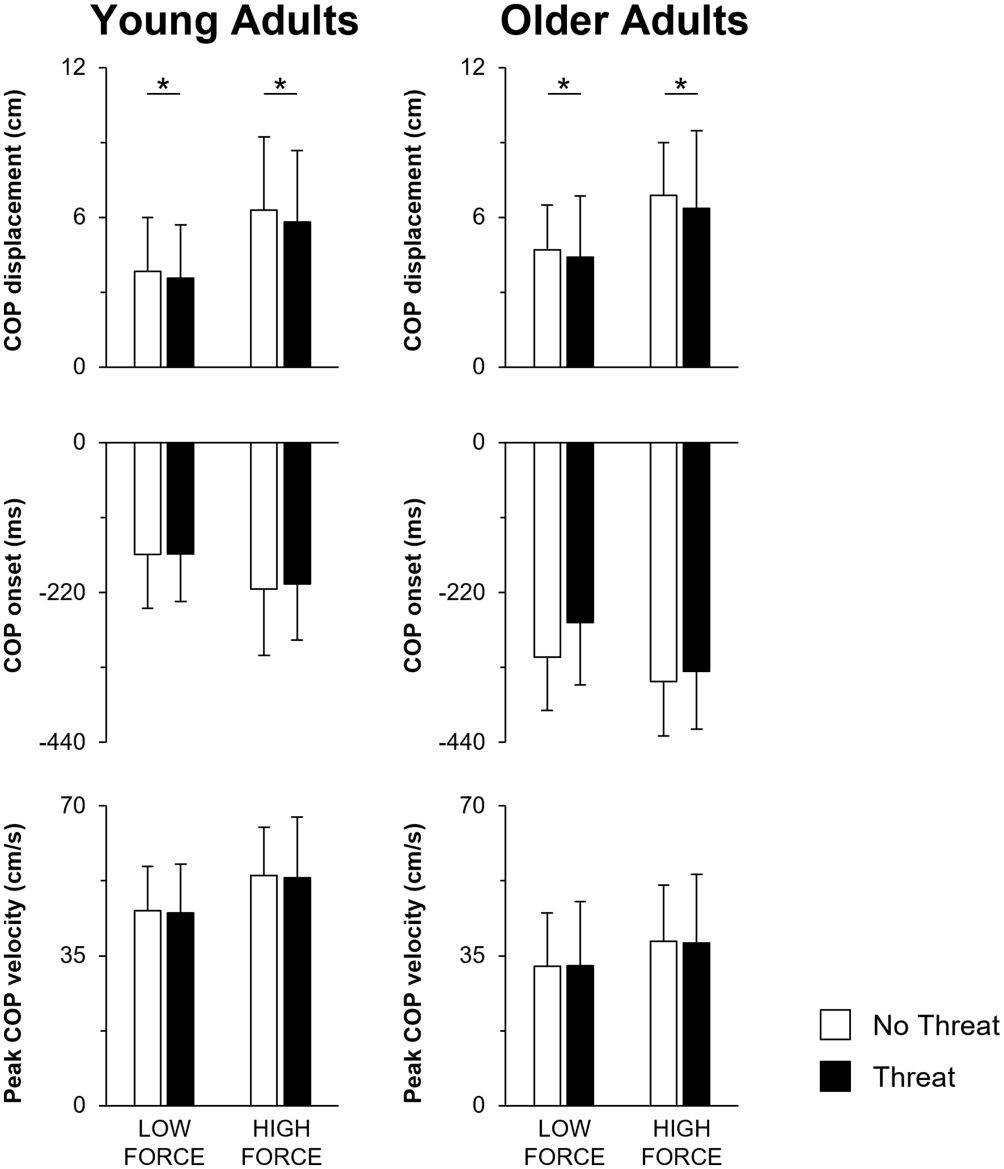
Mean COP displacement, COP onset and peak COP velocity when pulling with low or high force during the No Threat (white bars) and Threat (black bars) conditions. Data for the young and older adults are plotted on the left and right columns, respectively. Error bars represent one SD. For clarity, only significant threat effects (*p* < 0.05) are indicated by an asterisk (*). COP, center of pressure.

**Figure 4:**
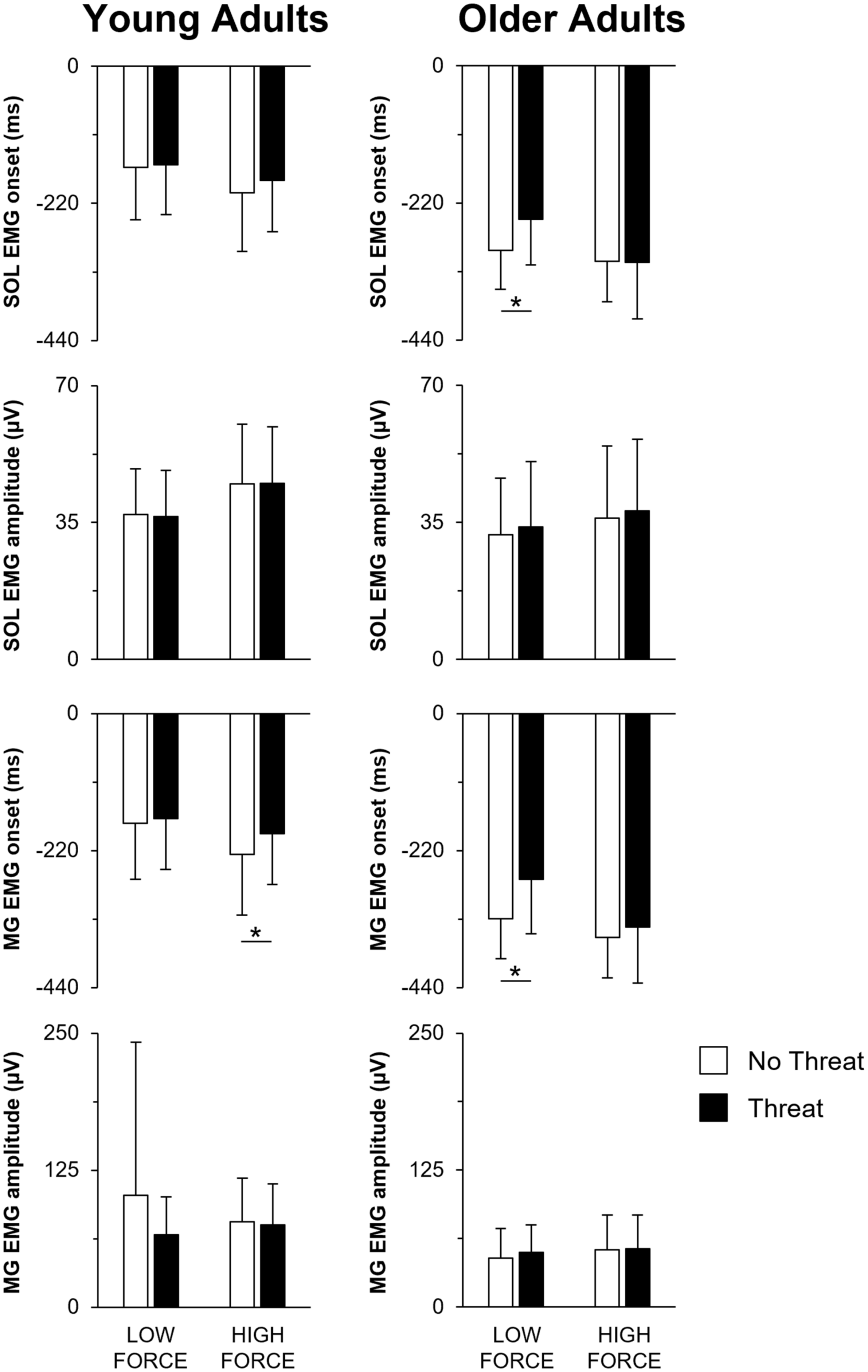
Mean SOL EMG onset, SOL EMG amplitude, MG EMG onset, and MG EMG amplitude when pulling with low or high force during the No Threat (white bars) and Threat (black bars) conditions. Data for the young and older adults are plotted on the left and right columns, respectively. Error bars represent one SD. For clarity, only significant threat effects (*p* < 0.05) are indicated by an asterisk (*). SOL, soleus; EMG, electromyography; MG, medial gastrocnemius.

When individuals pulled with greater force, the size of the preceding APA was larger in amplitude (Figures 3, 4). This was reflected by a 76.1 ± 72.9% increase in forward COP displacement (force main effect; F_1,25_ = 105.18; *p* < 0.001) and an 18.9 ± 11.6% increase in peak forward COP velocity (force main effect; F_1,25_ = 70.69; *p* < 0.001) from the low to high force condition across both young and older adults. Of these three dependent measures, only the COP velocity was also influenced by a main effect of age (F_1,25_ = 8.73; *p* = 0.007), with young adults generating a 39% larger COP velocity than the older adults. Lastly, the SOL EMG amplitude was influenced by an age × force interaction effect (F_1,25_ = 4.58; *p* = 0.042). A larger increase in SOL EMG amplitude between force conditions was observed in the young adults (22.7 ± 12.0% increase; *p* < 0.001) compared to the older adults (13.2 ± 13.7% increase; *p* = 0.009).

Not only did the size of the APA increase during the higher force condition, the APA was initiated earlier, leading to a longer duration APA (Figures 3, 4). Across both young and older adults, COP onset occurred earlier by 49.8 ± 32.4 ms in the high compared to the low force condition (force main effect; F_1,25_ = 59.80; *p* < 0.001). However, across all force and threat conditions, older adults initiated the APA earlier, as reflected by a 128.8 ms earlier COP onset, than the young (main effect of age; F_1,25_ = 18.62; *p* < 0.001).

#### 3.4.2. The effect of postural threat on APAs

A main effect of threat was only observed for COP displacement (F_1,25_ = 4.81; *p* = 0.038). When postural threat was present, young and older adults responded with a 3.8 ± 8.7 mm decrease in COP displacement (Figure 3). In contrast, both the SOL and MG EMG onset latencies were influenced by a three-way age × force × threat interaction effect (SOL: F_1,25_ = 14.21; *p* = 0.001; MG: F_1,25_ = 18.10; *p* < 0.001) (Figure 4). Post-hoc tests revealed that for the young adults, the presence of threat resulted in a 33.6 ± 43.1 ms delayed MG EMG onset latency during the high force condition. Postural threat did not influence the MG EMG onset latency during the low force condition (*p* = 0.314), or the SOL EMG onset latency at either force condition (*p* = 0.089) for the young adults. In contrast, postural threat resulted in a delay in the SOL (by 50.0 ± 46.2 ms; *p* = 0.008) and MG EMG onset latencies (by 63.6 ± 78.1 ms; *p* = 0.030) during the low force condition for the older adults.

## 4. Discussion

The purposes of this study were to determine whether the scaling of APAs with respect to force exertion is altered with aging or by changes in emotional state due to the presence of postural threat. The results of this study indicate that young adults and to a lesser extent, older adults scaled their APA, such that larger forces were preceded by larger and earlier APAs. Further, while some aspects of the APA were altered when postural threat was present, there were few interaction effects with pulling force. This would suggest that the scaling of APAs with increasing applied force was not largely affected by the presence of postural threat regardless of age.

### 4.1. Scaling of APAs with aging

During the No Threat condition, both young and older adults modulated the timing and amplitude of their APAs according to the force requirements of the upcoming pull. However, results from the regression analyses indicated that the degree to which APAs were scaled was reduced in older adults. Specifically, older adults exhibited smaller increases in COP velocity and SOL EMG amplitude as well as more homogenous SOL EMG onset latencies with increasing force. These results support the work of previous studies that have reported an under- and over-responding of APAs prior to movements involving maximal and submaximal forces, respectively, leading to a decreased scaling in older adults (Weeks, 1994; Blaszczyk et al., 1997; Lee et al., 2015).

Unlike the results of the regression analyses, the secondary analyses, which were performed only on data that were matched to the pulling force, yielded few significant age × force interaction effects. One might expect the secondary analyses to result in similar outcomes as the regression analyses since comparing the slope of the regression line between young and older adults should correspond to an age × force interaction effect. However, these discrepancies are likely the result of each method’s limitations. For example, since the regression analyses involved transforming the data based on each individual’s mean and deviation of pulling forces within each experimental condition, it is possible that the slope for each individual is derived from trials over a different range of pulling forces. Further, the slope represents a normalized difference rather than an absolute value. This may explain why older adults exhibited a more negative EMG onset latency, indicating an earlier EMG onset with increasing force, based on the secondary analyses but a less negative slope value for SOL or MG EMG onset latency according to the regression analyses. The flatter slope of the regression line would imply a less altered and not necessarily a delayed EMG onset with increasing force. Second, it must be noted that while the regression analyses considered all trials across the entirety of an individual’s range of pulling forces, the secondary analyses only included trials within two specific force bins (50–70 and 80–100 %MVC). While the use of two force bins ensured that trials with a similar pulling force were compared across subjects and threat conditions, it did result in fewer subjects and trials being included for analysis. It is possible that more significant effects would be observed if a larger separation (i.e., >10%) was incorporated between the low and high force conditions. This might be particularly important when examining how APAs differ with age, as older adults have been found to initiate APAs earlier or later than young adults depending on whether they are applying a submaximal or maximal force, respectively (Stelmach et al., 1990; Bleuse et al., 2006).

Regardless of the method of analyses, more prominent age-related differences in APAs might also have been observed if the study had incorporated a choice reaction time instead of a simple reaction time task (Inglin and Woollacott, 1988; Kubicki et al., 2011). When individuals can anticipate the APA requirements of an upcoming movement, as is the case with a simple reaction time task, and are given adequate time to prepare, an appropriately scaled APA is more likely to occur. In contrast, when there is uncertainty in the task requirements and individuals cannot pre-emptively prepare for a specific APA, as is the case with a choice reaction time task, then errors in scaling the APA amplitude or timing are more likely to occur. In this study, participants were informed of the trial’s force requirement a few seconds prior to the warning tone and had another 1–4 s before the “go” tone. This would have given participants, particularly the older adults, ample time to plan their APA prior to each pull. Furthermore, as noted by the dispersion of pulling forces, older adults pulled with a slightly smaller variance of forces (SD of 12.1% MVC) compared to the young adults (SD of 14.3 %MVC). This strategy would have reduced the need for older adults to regulate the size and timing of the APA from one trial to the next. Therefore, to maximize any age-related differences in the scaling of APAs, future studies should ensure that the same force levels are attained in all participants and provide the target force at a later time to minimize the time available for individuals to pre-select an appropriate force and corresponding APA.

### 4.2. The effect of postural threat on the scaling of APAs

This study presented a surface translation paradigm to induce postural threat. As intended, the presence of threat elicited an emotional and physiological response, in the form of an increase in physiological arousal, perceived anxiety and fear of falling, as well as a decrease in balance confidence. These changes in emotional state and physiological arousal resulted in several changes to the APA. Specifically, the secondary analyses indicated that regardless of force, both young and older adults reduced their COP displacement when postural threat was present. While this effect is opposite to what occurs with a heel raise in the presence of a potential postural perturbation (Phanthanourak et al., 2016; Bax et al., 2020), or with gait initiation performed at an elevated height (Ellmers et al., 2020), it supports the view that the effects of postural threat on APAs are dependent on the task and the type of threat being presented (Bax et al., 2020).

When the effect of postural threat on the scaling of APAs was examined, regression analyses revealed a significant age × threat interaction for COP displacement, but post-hoc analyses did not indicate any specific differences between groups or threat conditions. On the other hand, our secondary analyses revealed several changes that may alter the scaling of APA timing. Young adults activated the MG later when pulling at higher forces while under threat, which could result in a decrease to the scaling of MG EMG onset latency. In contrast, older adults demonstrated a delay in the SOL and MG onset latency during the lower forces while under threat. Both would serve to increase the scaling of the EMG onset latency with respect to pulling force. Thus, while there was some evidence that postural threat differentially alters the scaling of APAs between young and older adults, this cannot be conclusively stated given the inconsistent effects observed between analysis methods as well as the limited measures that were found to be affected by postural threat.

Several studies have reported that both young and older adults respond similarly to the presence of postural threat during quiet standing (Brown et al., 2006; Carpenter et al., 2006; Johannsson et al., 2017; Johnson et al., 2019). In contrast, greater threat-induced differences in APAs with age might be expected based on the work by Brown et al. (2002), who found that compared to young adults, older adults proactively respond to postural threat by walking in a more conservative manner. One possible reason for the smaller than expected change in APAs in this study may be that the older adults of this study perceived the surface translations to be less threatening than the young. Smaller changes in anxiety, fear and balance confidence were reported by the older compared to the young adults. Our results may have also been influenced by the participant characteristics, as the older adults reported high levels of balance confidence (ABC scores >95%). Larger age- and threat-related differences on the scaling of anticipatory postural responses may arise when testing individuals with higher anxiety or lower balance confidence instead.

The minimal age-related differences in APAs with postural threat may have also been the result of young and older adults adopting different pulling strategies when threatened. For example, older adults responded to threat by pulling harder (mean force of 71 %MVC vs. 63 %MVC) and with a smaller variance of forces (SD of 11 %MVC vs. 13% %MVC) than when threat was absent. It is possible that the stronger pulls were due to a greater activation of the prefrontal cortical areas elicited by an increased state of emotional arousal (Schmidt et al., 2009) but if this were the case, a similar effect would be expected in the young adults. But for these younger individuals, no differences in pulling force were observed between the No Threat and Threat conditions. Thus, pulling with a more central (i.e., closer towards the midpoint between the lowest and highest possible target force) and constant force regardless of the actual instructed force may have been a deliberate strategy adopted by older adults. Not only would such a strategy reduce the need to modulate the size and timing of the APA for each trial, it would allow older adults to focus more of their attention towards the potential perturbation even though it may be at the expense of not generating the most appropriate APA for each upcoming pull.

Aside from issues described previously, two other limitations should be considered. First, all participants completed the No Threat condition before the Threat condition since prior experience with postural threat can influence postural control even in the absence of the threat (Adkin et al., 2000). However, since the conditions were not counterbalanced between participants, the possibility of an order effect must be acknowledged. Second, although increases in feelings of anxiety and fear of falling were observed with postural threat, it is possible that over time, some of the participants may have habituated to the inflexible characteristics of the surface translations. Johnson et al. (2019) reported that by the 24th exposure to the threat of perturbation, there were significant reductions in anxiety, arousal, attention focus, along with some changes in standing balance measures. Since this study included 45 Threat trials, this may have resulted in some participants perceiving the postural threat to be less threatening as the experimental trials progressed and could have reduced any potential differences in APAs between the No Threat and Threat conditions. Although our exploratory analyses did not reveal any consistent differences in the scaling of the APAs between the first and last quartile of the Threat trials, future protocols might consider incorporating fewer Threat trials or eliminating trials where a participant is no longer feeling threatened (e.g., having an EDA that has returned to the baseline No Threat level). This would ensure that any changes between the No Threat and Threat groups of trials would truly be due to a threat-related response (i.e., increased physiological arousal, anxiety, and fear of falling).

## 5. Conclusion

In conclusion, the results of this study indicate that postural threat alters aspects of the APA associated with a pulling task. Although postural threat resulted in some differences in how young and older adults scale their APAs according to the size of the upcoming movement, further examination and analyses are required to confirm this effect. Larger differences might be revealed through slight alterations in the experimental protocol or by testing older adults with a history of falling or those with impaired balance ability.

## Data availability statement

The raw data supporting the conclusions of this article will be made available by the authors, without undue reservation.

## Ethics statement

The studies involving humans were approved by Brock University Health Science Research Ethics Board. The studies were conducted in accordance with the local legislation and institutional requirements. The participants provided their written informed consent to participate in this study.

## Author contributions

AP: Writing – original draft, Writing – review & editing, Conceptualization, Investigation, Formal Analysis. AA: Writing – review & editing, Conceptualization. MC: Writing – review & editing, Conceptualization. CT: Writing – original draft, Writing – review & editing, Conceptualization, Formal Analysis, Supervision.
